# Seeing the Stricture Clearly: Independent Determinants of Sonourethrography Precision in Urethral Stricture Disease

**DOI:** 10.3390/jcm14134453

**Published:** 2025-06-23

**Authors:** Kevin Miszewski, Jakub Krukowski, Laura Miszewska, Jakub Kulski, Roland Stec, Katarzyna Skrobisz, Marcin Matuszewski

**Affiliations:** 1 Department of Urology, Medical University of Gdańsk, 17 Smoluchowskiego St., 80-214 Gdańsk, Poland; 2 Faculty of Medicine, Medical University of Gdańsk, 17 Smoluchowskiego St., 80-214 Gdańsk, Poland; 3 Department of Radiology, Medical University of Gdańsk, 17 Smoluchowskiego St., 80-210 Gdańsk, Poland

**Keywords:** urethral stricture, sonourethrography, imaging accuracy, urethroplasty, spongiofibrosis, predictive factors

## Abstract

**Background**: Urethral stricture disease involves fibrotic scarring that narrows the urethral lumen and can occur at any site. Sonourethrography (SUG) is increasingly used because it depicts both luminal anatomy and periurethral fibrosis, yet little is known about patient or lesion features that influence its diagnostic performance. **Methods**: We conducted a prospective single-center study of 170 men who underwent SUG before anterior urethroplasty between May 2016 and May 2021. Anthropometric data, comorbidities, and detailed ultrasonographic measurements were recorded and compared with intra-operative findings, which served as the reference standard. Accuracy was analyzed with Wald chi-square testing and Spearman correlation. **Results**: SUG length estimates matched intra-operative measurements in 139/170 strictures (81.8%). Length accuracy was higher in patients ≥ 60 years (89.2% vs. 77.0%, *p* = 0.03) and in those with type 2 diabetes (92.3% vs. 80.9%, *p* = 0.02) in conditions associated with pronounced spongiofibrosis that enhances echo contrast. Among stricture-specific factors, proximal location (63.6% vs. 84.5%, *p* = 0.01) and complete luminal occlusion (68.8% vs. 84.8%, *p* = 0.02) reduced precision, largely because deeper anatomy and absent saline flow hinder acoustic delineation. The Chiou ultrasonographic grade was the strongest determinant of performance; higher grades yielded clearer margins and better length estimation (*p* < 0.001). **Conclusions**: SUG is a reliable bedside technique for assessing anterior urethral strictures, but its accuracy varies with age, diabetes status, stricture site, degree of occlusion, and fibrosis grade. Recognizing these determinants allows clinicians to judge when SUG alone is sufficient and when complementary imaging or heightened caution is warranted. The findings support tailored imaging protocols and underscore the need for multi-center studies that include operators with diverse experience to confirm generalisability.

## 1. Introduction

Urethral stricture develops due to a process of fibrosis and cicatrix formation within the urethral mucosa and surrounding tissues [1,2]. Lesions can occur anywhere from the external meatus to the bladder neck and may follow trauma, infection, iatrogenic instrumentation, or chronic inflammatory disorders [3,4]. As fibrosis continues to contract, it progressively constricts the urethral lumen, leading to obstructive lower urinary tract symptoms. Patients report a weak or intermittent stream, hesitancy, straining, and a persistent sensation of incomplete bladder emptying—symptoms that mirror the escalating outflow resistance created by the fibrotic segment [5]. Apart from obstructive voiding, urethral strictures markedly undermine everyday quality of life, with studies reporting urinary tract infections in 41% of patients and urinary incontinence in 11% [6]. Once this impairment becomes clinically significant, the treatment strategy is guided by both the expected durability of symptom relief and the anatomical characteristics of the stricture as revealed by imaging. Factors such as stricture length, location, and the extent of surrounding fibrosis are essential in determining whether endoscopic management is appropriate or if definitive reconstructive surgery should be pursued. Endoscopic measures such as single-session dilatation or direct vision internal urethrotomy (DVIU) remain appealing for their simplicity and feasibility under regional anesthesia; however, their long-term efficacy is limited. Even among carefully selected patients with short, primary bulbar strictures, stricture-free rates at one year rarely exceed 60%, and outcomes deteriorate markedly with repeated procedures [7]. Definitive urethroplasty is, therefore, offered sooner today than it was a decade ago, and the operation chosen is dictated by stricture anatomy [8]. For short (<2 cm) bulbar segments, excision and primary anastomosis, which now include modern non-transecting variants that preserve the corporal blood supply, consistently achieve patency rates above 90% with minimal impact on sexual function [9]. When segments are long or penile, single-stage dorsal or ventral buccal–mucosa graft urethroplasty is preferred, and a recent multicentre series with a median 7-year follow-up reported 88% success and very low decision regret [10]. By contrast, strictures driven by lichen sclerosus, prior radiation. or pan-urethral fibrosis usually requires staged reconstruction or, when reconstruction is unfeasible, perineal urethrostomy, as recommended in the 2023 EAU Guidelines [11]. Accurate imaging is what links these therapeutic decisions to the individual patient. For decades, voiding cystourethrography (VCUG) and urethrocystoscopy have been used as initial imaging tools in stricture evaluation [12]. They are readily available, inexpensive, and provide immediate information on stricture site and length, including reliable assessment of the posterior urethra. Their chief drawback is that they visualize only the urethral lumen; the peri-urethral tissues and degree of illustrating the extent of corpus spongiosum fibrosis (spongiofibrosis) cannot be assessed. Comparative studies have shown that VCUG may underestimate true stricture length [13,14]. Both patient and operator are additionally exposed to ionizing radiation during the study, which becomes a more consequential issue when repeat imaging is required. These limitations have motivated clinicians to seek more comprehensive imaging methods. Magnetic resonance urethrography (MRU) supplies high-resolution, multiplanar views with excellent soft-tissue contrast, depicting both the urethral lumen and surrounding spongiofibrosis while completely avoiding ionizing radiation [15]. Routine adoption of MRU, however, remains restricted by cost and access, so many centers still rely on a complementary modality that can be delivered at the bedside and repeated without risk. High-resolution ultrasound meets these requirements. Sonourethrography (SUG), first described by McAninch in 1988 [16], uses a 7–12 MHz linear probe to outline the urethral lumen and the surrounding corpus spongiosum in real time, providing the very information that fluoroscopy and even cystoscopy cannot capture [17,18]. Over the past three decades, multiple comparative series have confirmed that SUG measures stricture length more accurately than retrograde urethrography and grades spongiofibrosis with a level of detail matched only by intra-operative inspection [19,20,21,22]. Its diagnostic performance, however, is highly dependent on operator expertise and on image-optimization factors such as transducer pressure and instillation technique; the 2023 EAU Guidelines on urethral stricture disease specifically highlight that SUG should be carried out by clinicians with dedicated ultrasound training because its accuracy is strongly operator dependent [23]. Limitations of the technique include lower sensitivity for detecting bulbar urethral strictures, the requirement for intra-urethral distension (and therefore local anesthesia), and the fact that specialized training is not yet widespread, all of which constrain routine adoption.

Despite its demonstrated effectiveness, surprisingly few studies have explored which patient- or disease-specific factors independently influence the accuracy of SUG. Addressing that gap is clinically relevant because inaccurate length or grade estimations can misguide the choice between endoscopic treatment and definitive urethroplasty. The present prospective single-center study, therefore, set out to identify anthropometric, comorbid, and ultrasonographic variables that influence SUG accuracy in anterior urethral strictures, using intra-operative measurements as the reference standard. By clarifying when SUG can be relied upon and when caution is warranted, we hope to refine imaging protocols and improve surgical planning for patients with this challenging condition.

## 2. Materials and Methods

### 2.1. Study Design and Population

This prospective, single-center study was conducted between May 2016 and May 2021 at a tertiary referral center specializing in urethral stricture disease. The study protocol was approved by the local Independent Ethics Committee, and written, informed consent was obtained from each patient prior to participation. Initially, 216 adult Caucasian men (≥18 years) with anterior urethral strictures documented on retrograde plus voiding cystourethrography (VCUG), the modality endorsed by the current European Association of Urology Guidelines for initial imaging, were considered for inclusion.

The exclusion criteria were:

Clinically significant meatal stenosis that prevented placement and balloon anchorage of a 14 Ch Foley catheter in the fossa navicularis, a prerequisite for saline instillation during SUG. If such stenosis co-existed with a more proximal stricture, the patient was still excluded for this technical reason. Balanitis xerotica obliterans (BXO) in itself was not an exclusion criterion; men with BXO were included, provided the meatus could accommodate the catheter.

1. Complete urethral obliteration in which the proximal edge of the stricture could not be identified on ultrasound,
2. Treatment modalities other than open reconstructive urethroplasty (e.g., patients opting out of surgery or managed with dilation only).

After applying these criteria, 170 patients remained eligible and were included in the final analysis.

### 2.2. Sonourethrography Protocol

All SUG procedures were carried out by a single experienced urologist. Urethral ultrasonography was performed with a Flex Focus 800 ultrasound system (BK Medical, Peabody, MA, USA) equipped with a 9–18 MHz linear-array transducer and running console software version 4.86.12256.26.

Patient Preparation:

3. The external urethral meatus was disinfected.
4. Catheter placement: A 14 Ch Foley catheter was introduced gently into the urethra. The Foley balloon was filled with approximately 2 mL of normal saline and secured within the fossa navicularis to prevent fluid leakage.
5. Saline distension: Next, the urethra was infused with 0.9% normal saline solution.

**Figure 1:**
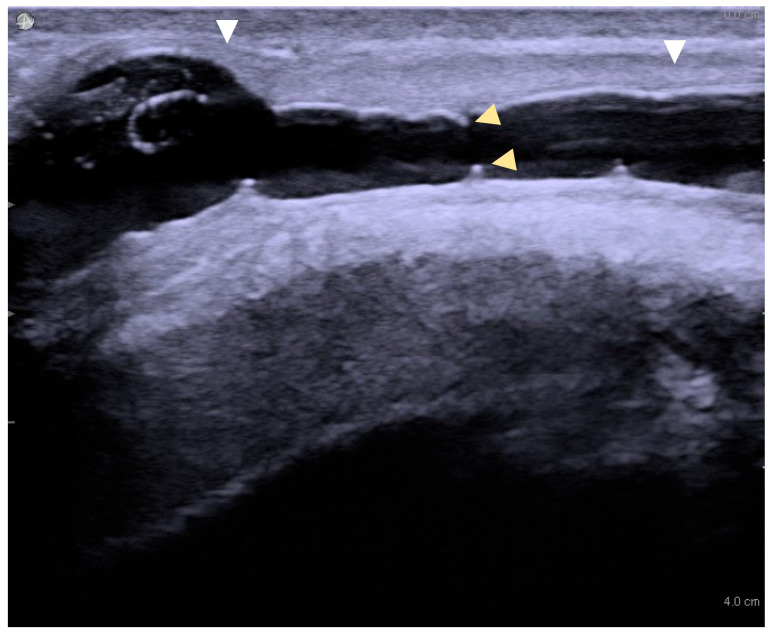
Long-segment urethral stricture with spongiofibrosis on sonourethrography. White arrowheads mark the proximal and distal stricture margins (length ≈ 3.2 cm); yellow arrowheads indicate the luminal diameter (≈4 mm).

Image acquisition: Ultrasound images were captured in both transverse and longitudinal planes. A normal urethra appears as a uniform, echo-free area, measuring approximately 8–12 mm in diameter, depending on the urethral segment. Any region that failed to distend with saline infusion was recorded as a stricture site. Spongiofibrosis was identified by hyperechoic areas within the corpus spongiosum (Figure 1).

To standardize assessment, stricture grade was recorded using a five-point Chiou scale, with higher values indicating increasing degrees of luminal compromise and fibrosis. As a surgical “gold standard”, intraoperative measurements recorded during open urethroplasty served as the reference for stricture characteristics. Agreement between SUG and intra-operative stricture length was considered accurate when the absolute discrepancy was ≤5 mm, a cut-off first formalized by Choudhary et al. [18] and subsequently applied by Krukowski et al. [13] because discrepancies larger than this occasionally alter operative strategy, whereas smaller differences seldom do.

### 2.3. Data Collection

Demographic and anthropometric data—age, height, weight, and body mass index (BMI)—were collected. Comorbidities included hypertension (NT), diabetes mellitus, and smoking status. The presence of bacteriuria was documented at the time of admission. Stricture-specific variables were recorded, including the location of the stricture (e.g., penile, bulbar, or more proximal segments), length of the stricture (as measured by SUG and confirmed intraoperatively), degree of luminal narrowing (classified as partial flow preserved, critical narrowing, or complete occlusion of the urethral lumen), etiology (e.g., traumatic, iatrogenic, inflammatory, or idiopathic), previous urethral interventions (dilation, internal urethrotomy, and prior urethroplasty).

Additionally, ultrasonographic variables noted during SUG included the stricture grade (1–5), the distance from the skin to the corpus spongiosum, and the distance from the skin to the proximal stricture edge.

### 2.4. Statistical Analysis

Statistical analysis was conducted using Statistica 13 (StatSoft Inc., Tulsa, OK, USA). Normality and variance were evaluated using the Shapiro–Wilk test. Variables with a non-normal distribution are expressed as median with inter-quartile range (IQR) and full range; normally distributed variables are presented as mean ± standard deviation (SD). Correlations between variables were analyzed using Spearman’s correlation coefficient. Continuous variables were compared using the independent *t*-test or Mann–Whitney U-test, depending on the distribution. The Wald chi-square test was used to evaluate the significance of each independent variable in determining the accuracy of SUG findings. A *p*-value of <0.05 was considered statistically significant.

## 3. Results

### 3.1. Patient Demographics and Stricture Characteristics

**Figure 2:**
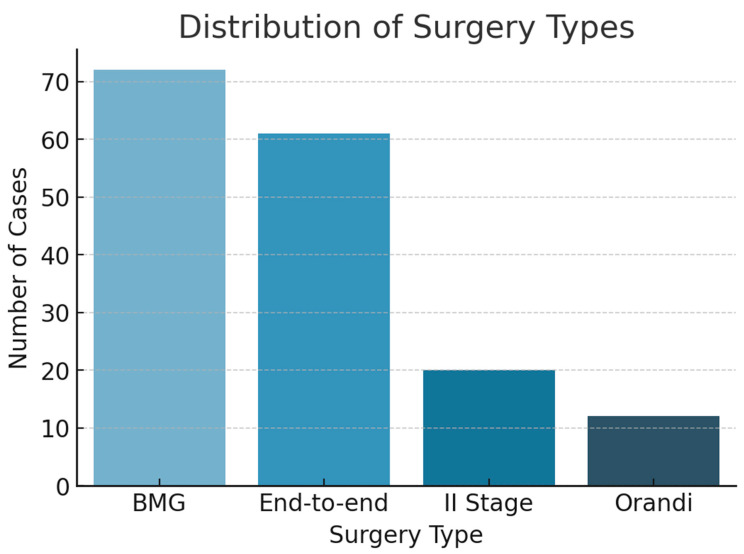
Distribution of surgery types.

Following exclusions, 170 men fulfilled the inclusion criteria and underwent SUG prior to open reconstructive urethroplasty. Table 1 summarizes the demographic and clinical characteristics. The mean age was 52.6 years (range: 19–79). Bulbar strictures were the most common location, consistent with prior literature [3,4]. The most frequent cause of urethral injury was traumatic (e.g., straddle injuries), followed by iatrogenic, inflammatory, and idiopathic etiologies. Regarding surgical history, most patients had undergone at least one prior intervention: 122 men (71.8%) had received an optical internal urethrotomy (UIO), 32 (18.8%) had undergone a previous urethroplasty, and overall 135 patients (79.4%) had a record of any previous urethral surgery. Table 2 compares stricture-length estimates obtained with each modality. VCUG yielded the shortest median length at 12 mm [IQR 5–20] (range 0–90), SUG measured 26 mm (20–34) (3–80), and intra-operative assessment confirmed a median of 30 mm (20–40) (2–120). Mean values showed the same hierarchy—15.8 ± 14.7 mm for VCUG, 28.4 ± 13.5 mm for SUG, and 30.5 ± 17.8 mm intra-operatively. Mucosal graft urethroplasty was the most common surgical technique performed Figure 2.

Among the anthropometric variables, only age significantly influenced SUG length accuracy (χ^2^ = 4.68, *p* = 0.03). Neither height nor weight (nor BMI) demonstrated a statistically significant effect.

### 3.2. Comorbidities and Impact on Imaging

Of the recorded comorbidities, diabetes mellitus was the only variable that significantly affected SUG quality (χ^2 = 5.05, *p* = 0.02). Patients with diabetes often exhibited more pronounced spongiofibrosis, which appeared to enhance the sonographic contrast and improve the delineation of stricture boundaries. Hypertension and dyslipidemia did not significantly alter imaging quality. Bacteriuria and smoking history were also evaluated but did not show a statistically relevant impact on measurement accuracy.

### 3.3. Stricture-Specific Variables

**Figure 3:**
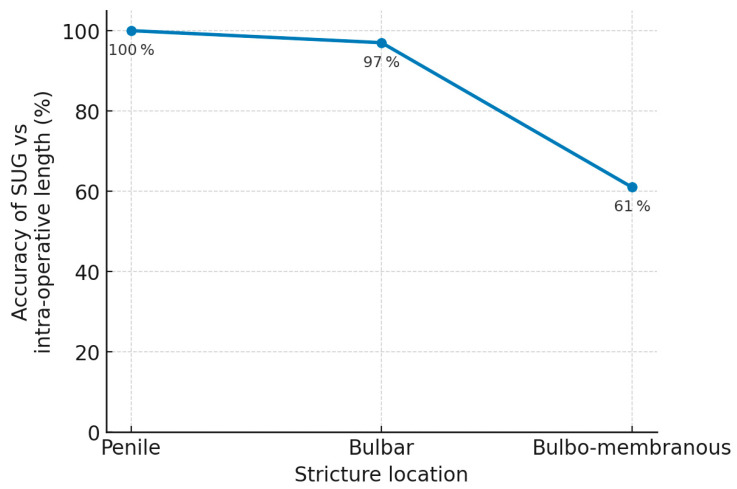
Effect of stricture location sonourethrography accuracy.

Analysis of stricture location revealed that more proximal strictures were associated with lower SUG accuracy (χ^2^ = 8.55, *p* = 0.01), likely due to challenges in probe positioning and deeper anatomical location Figure 3. The degree of luminal narrowing also correlated with diminished accuracy (χ^2^ = 7.37, *p* = 0.02). Complete urethral occlusions posed the greatest difficulty in distinguishing stricture boundaries sonographically.

Neither the number nor the type of prior endoscopic interventions significantly affected the quality of SUG.

### 3.4. Ultrasonographic Variables

**Figure 4:**
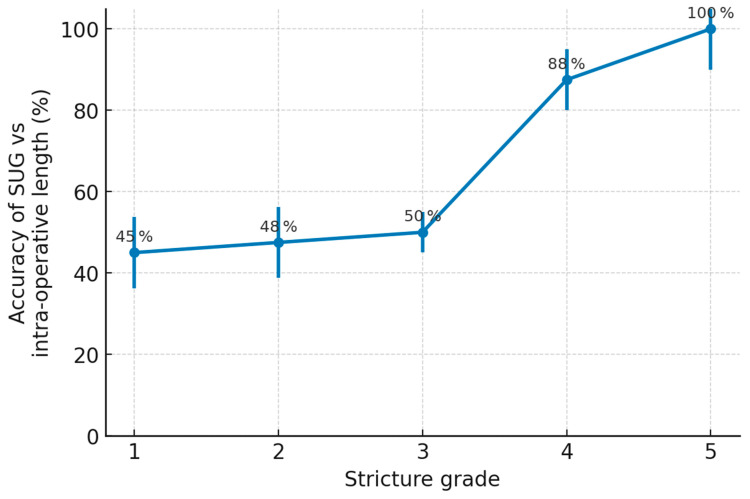
Impact of Chiou stricture grade on the accuracy of sonourethrography.

Among the ultrasonographic variables, stricture grade—assessed using the five-point Chiou classification—was the dominant factor influencing imaging accuracy (χ^2^ = 90.55, *p* < 0.001). Strictures of higher grade (indicating greater fibrosis and more severe luminal compromise) were paradoxically easier to delineate because the fibrotic tissue produced a clearer hyperechoic signature compared to mild or moderate strictures Figure 4.

### 3.5. Intraoperative Correlations

During open reconstructive urethroplasty, intraoperative measurements confirmed that SUG reliably estimated stricture length and spongiofibrosis in most patients. However, in complete lumen closure, the accuracy decreased markedly (*p* = 0.02). This limitation underscores the challenge of imaging when no fluid can pass across the stricture site to provide acoustic contrast.

## 4. Discussion

For over three decades, sonourethrography has been an essential modality for the preoperative evaluation of anterior urethral strictures [16,17,18,19]. Compared to CUG, SUG offers superior spatial resolution for defining stricture length and the extent of spongiofibrosis, providing invaluable details for reconstructive planning [18,22]. Yet, the influence of patient factors on SUG accuracy has rarely been explored.

In our study, we identified several factors influencing the accuracy of SUG. Notably, patient age emerged as a significant anthropometric variable. Older patients may exhibit increased tissue fibrosis or reduced urethral compliance, enhancing ultrasound contrast and facilitating stricture visualization. This finding aligns with studies suggesting age-related changes in tissue structure impact imaging performance [24]. Diabetes mellitus was another comorbidity associated with improved SUG accuracy. Patients with diabetes often present with more pronounced spongiofibrosis, which appears as hyperechoic areas on ultrasound, delineating stricture margins more clearly [25]. This observation is supported by previous work showing a link between poor glycemic control and fibrosis development [24]. Neither hypertension, dyslipidemia, nor smoking status significantly altered imaging quality.

Stricture-specific attributes also played a role. Proximal urethral strictures were associated with lower imaging accuracy, likely due to anatomical depth and suboptimal probe positioning [26]. Complete luminal occlusion further compromised SUG precision due to the absence of fluid passage and resultant acoustic shadowing. Among ultrasonographic variables, stricture grade (Chiou scale) was the strongest determinant of imaging performance. Higher-grade strictures, marked by dense fibrosis and severe narrowing, were paradoxically easier to delineate due to their prominent hyperechoic signature [21]. Our observations reinforce the notion that while SUG provides exceptional detail, it is not without limitations. Situations with severe luminal occlusion or anatomically challenging stricture locations can compromise accuracy. Despite these limitations, the capacity of SUG to assess spongiofibrosis remains unmatched by conventional fluoroscopic methods [17,18,21,22].

Technical innovations promise to offset some of these shortcomings. Shear wave elastography differentiates scarred spongiosum from healthy tissue by quantifying stiffness; in a 2024 prospective study, elasticity ratios correlated closely with histological fibrosis and improved length estimation in difficult cases [27]. Pilot work with contrast-enhanced ultrasound shows that microbubble perfusion highlights areas of vascularised scar and increases agreement on stricture boundaries, particularly in recurrent disease [22]. Beyond single-plane imaging, artificial-intelligence assisted three-dimensional reconstruction can now segment the urethral lumen and surrounding scar within minutes and produces length measurements that mirror intra-operative findings within 2 mm [28].

Operator dependency remains the principal limitation of SUG. A 2023 review of 18 prospective series reported inter-observer kappa values ranging from 0.42 to 0.88 but noted that agreement rose markedly after structured hands-on training and the use of standardized acquisition protocols [29]. Our study, performed by a single expert, was not designed to address variability, yet it underscores the need for wider training programmes if SUG is to move beyond tertiary centres. Prospective trials have already shown that adding SUG to retrograde urethrography changes the planned surgical approach in up to 35 percent of cases, mainly by identifying longer segments of spongiofibrosis that require grafting rather than excision and anastomosis [30].

This study has several important limitations. First, it represents the experience of a single center, and, importantly, all 170 examinations were performed and interpreted by the same highly experienced operator. While this eliminated inter-observer variability, it also narrows external validity; the diagnostic accuracy reported here may not be reproducible in lower-volume settings or among clinicians with less ultrasound training. Second, every SUG examination was performed by one highly experienced urologist; the absence of less-experienced operators prevents any assessment of inter-observer variability or generalisability across different skill levels. Third, although intra-operative visual inspection is commonly used by other investigators, it is an imperfect benchmark for grading spongiofibrosis [31]. Fourth, although all examinations followed the same local protocol, the study did not evaluate how variations in catheter caliber, instilled volume, transducer pressure, or patient positioning might influence accuracy. Because these factors differ among centers, a consensus acquisition would be essential for wider reproducibility of SUG results. Fifth, the surgeon who acquired the SUG images also performed the urethroplasty and documented the reference measurements; the absence of operator blinding may have favored concordance between modalities, and future studies should separate image acquisition from surgical assessment or include an independent reviewer. To overcome these constraints and deepen understanding of urethral stricture disease, large-scale multicentre studies that incorporate multiple operators of varying experience and histopathological correlation are now required.

## 5. Conclusions

Sonourethrography proved to be a dependable, readily repeatable technique for characterizing anterior urethral strictures, with measurements that correlated closely with intraoperative findings in the vast majority of cases. Its precision, however, is not uniform across all patients. We identified five independent determinants: advanced age, diabetes mellitus, proximal stricture location, complete luminal occlusion, and high ultrasonographic (Chiou) grade, all of which systematically affect imaging accuracy. Awareness of these factors allows clinicians to anticipate when SUG is most likely to yield reliable data and when supplementary imaging or heightened caution in surgical planning is warranted. Although our single-center design and reliance on a single expert operator limit generalisability, the results underline the value of integrating patient-specific and lesion-specific variables into imaging protocols. Multicentre studies involving operators of differing experiences and incorporating histopathological correlation are now needed to validate these findings and refine sonourethrography-based algorithms for the definitive management of urethral stricture disease.

## Figures and Tables

**Table 1:** Demographic and Clinical Characteristics of the Study Population (N = 170).

Characteristic	Value
Age (years), median (IQR) (range)	59 (41–67) (18–82)
Height (cm), median (IQR) (range)	176 (170–180) (156–196)
Weight (kg), mean ± SD	84.3 ± 13.7
BMI (kg/m²), mean ± SD	27.4 ± 4.1
Diabetes mellitus (Type II), n (%)	13 (8.4)
Hypertension, n (%)	58 (36.9)
Current smokers, n (%)	32 (20.7)
Bacteriuria, n (%)	70 (44.9)
Stricture location, n(%)	Penile: 65 (38.3) Bulbar: 74 (43.5) Bulbo-membranous: 31 (18.2)
Previous urethral surgery, n (%):	Optical internal urethrotomy UIO:122 (71.8) Urethroplasty: 32 (18.8) Any previous urethral surgery: 135 (79.4)
Surgical technique, n (%):	Mucosal graft (BMG): 72 (43.6) End-to-end repair: 61 (37.0) Two-stage repair: 20 (12.1) Orandi: 12 (7.3)

**Table 2:** Comparison of Stricture-Length Measurements Obtained by Each Modality and Intra-operative Findings.

Modality	Median (IQR) (Range), mm	Mean ± SD, mm
Retrograde + voiding cystourethrography	12 (5–20) (0–90)	15.8 ± 14.7
Sonourethrography	26 (20–34) (3–80)	28.4 ± 13.5
Intra-operative measurement	30 (20–40) (2–120)	30.5 ± 17.8

## Data Availability

The data presented in this study are available on request from the corresponding author.
